# Excited States and Photodebromination of Selected Polybrominated Diphenyl Ethers: Computational and Quantitative Structure—Property Relationship Studies

**DOI:** 10.3390/ijms16011160

**Published:** 2015-01-06

**Authors:** Jin Luo, Jiwei Hu, Xionghui Wei, Lingyun Li, Xianfei Huang

**Affiliations:** 1 Guizhou Provincial Key Laboratory for Information System of Mountainous Areas and Protection of Ecological Environment, Guizhou Normal University, Guiyang 550001, China; E-Mails: luojin@gznu.edu.cn(J.L.); lingyunli1989@126.com (L.L); hxfswjs@gznu.edu.cn (X.H.); 2 Department of Applied Chemistry, College of Chemistry and Molecular Engineering, Peking University, Beijing 100871, China; E-Mail: xhwei@pku.edu.cn

**Keywords:** Polybrominateddiphenyl ethers, theoretical study, excited states, photodebromination, quantitative structure-property relationship, artificial neural network

## Abstract

This paper presents a density functional theory (DFT)/time-dependent DFT (TD-DFT) study on the lowest lying singlet and triplet excited states of 20 selected polybrominateddiphenyl ether (PBDE) congeners, with the solvation effect included in the calculations using the polarized continuum model (PCM). The results obtained showed that for most of the brominated diphenyl ether (BDE) congeners, the lowest singlet excited state was initiated by the electron transfer from HOMO to LUMO, involving a π–σ* excitation. In triplet excited states, structure of the BDE congeners differed notably from that of the BDE ground states with one of the specific C–Br bonds bending off the aromatic plane. In addition, the partial least squares regression (PLSR), principal component analysis-multiple linear regression analysis (PCA-MLR), and back propagation artificial neural network (BP-ANN) approaches were employed for a quantitative structure-property relationship (QSPR) study. Based on the previously reported kinetic data for the debromination by ultraviolet (UV) and sunlight, obtained QSPR models exhibited a reasonable evaluation of the photodebromination reactivity even when the BDE congeners had same degree of bromination, albeit different patterns of bromination.

## 1. Introduction

Polybrominateddiphenyl ethers (PBDEs) are organobromine compounds that are widely used as an additive flame retardant in polymers and are thus now widespread in the environment [1,2,3,4,5]. Due to their environmental persistency, lipophilicity, and potential toxicity (such as endocrine disruption, mutagenic harm to animals), PBDEs have aroused increasing concerns from both environmental chemists and biologists [6,7,8,9,10,11,12,13]. Furthermore, highly toxic products, such as polybrominated dibenzofurans (PBDFs) and polybrominateddibenzo-p-dioxins (PBDDs), might be generated by the combustion or photolysis of PBDEs [14,15,16].

Recently, photochemical debromination is recognized as a key transformation pathway of highly brominated PBDEs [17]. According to an estimation of the removal rates of PBDEs from the lower troposphere, the photolysis accounted for more than 90% of the removal of gas-phase congeners [18]. The direct photochemical debromination is also known to be an important process for the transformation of highly brominated BDEs to lower brominated congeners in the environment. In addition, the photodebromination for PBDEs is a relatively rapid and efficient remediation approach. For example, the photodebromination rate of BDE-209 [19] is approximately ten times higher than the reductive debromination rate by nanoscale zero-valent iron [20], and approximately equal to the debromination rate with the use of smectite clay-templated subnanoscale zero-valent iron [21]. Remediation of PBDEs via the photolysis approach with sunlight would be promising since solar energy is considered to be a clean and renewable energy. The current interpretation of these photolytic processes is mainly based on the excitation of PBDEs, by which the formed singlet or triplet excited states can undergo C–Br cleavage of PBDEs [22,23,24], as shown in Equations (1) and (2).

(1) PBDEs+UV→PBDEs (singlet excited state)→PBDEs (triplet excited state)

(2) PBDEs (singlet excited state or triplet excited state)→debromination

Excitation energy is highly related to the photolysis reactions. For example, it was found that the lowest singlet vertical excitation energy rather than hydrogen-donating efficiency and electron-donating efficiency of solvents contributes to relative resistance of bromine removal of BDE-209 [25]. Several quantitative structure-property relationship (QSPR) studies were previously carried out to investigate the photodebromination rates and quantum yields of PBDEs [26,27]. However, in these QSPR models, the excitation energy was not included as the molecular descriptors, and the studies on the relationship between the excitation states and the photodebromination of PBDEs are still insufficient and incomplete [28]. It is generally believed that, by spin-orbit coupling, halogenated aromatic hydrocarbons can be efficiently relaxed to triplet levels [29]. Triplet excited states are important in many kinds of photo-induced reactions due to the relatively long lifetimes bestowed on excited triplet species [30,31]. Hitherto, only limited studies on the debromination of PBDEs’ triplet excited states were reported. In addition, the QSPR studies conducted were only devoted to the photodebromination rate constants of PBDEsbyultraviolet (UV), but the QSPR studies regarding the photodebromination rate constants of PBDEs by sunlight have not yet been reported.

Eriksson* et al.* [19] and Wei* et al.* [32] have investigated the debromination of several BDE congeners, covering a range of different bromination patterns and degrees. To better investigate the relationship between the excited states and the photodebromination of PBDEs, 20BDE congeners were selected from their reported experiments for a computational study in the present work. The vertical lowest-lying singlet and triplet excited states were investigated by density functional theory (DFT)/time-dependent density functional theory (TD-DFT) calculations.

In addition, linear and nonlinear models were employed for a quantitative structure-property relationship (QSPR) study based on the reported photodebromination rate constants of PBDEs by UV [19] and by sunlight [32]. In traditional multiple linear regression methods, a dataset may contain redundant descriptors, and the correlations observed may be chance correlations. Moreover, the relationship between reaction rates and its parameter factors is in general considered nonlinear by nature. Therefore, we choose the two linear based models (partial least squares regression (PLSR) and principal component analysis-multiple linear regression (PCA-MLR)) and one nonlinear model (back propagation artificial neural network (BP-ANN)) to resolve the inter correlation problem of the descriptors and to remove the limitation of the assumption of a linear relationship between the reaction rate and descriptors, respectively. We also considered and studied the over fitting problems encountered when using the BP-ANN methods since this might be useful for significantly improving the performance of the BP-ANN model.

## 2. Results and Discussion

### 2.1. The Lowest-Lying Singlet Excitations for Selected Brominated Diphenyl Ether (BDE) Congeners

The lowest excited state (S_1_) is crucial to the mechanism explanation of the photochemical degradation of PBDEs [25,28], and S_1_ is the excited state which can be achieved not only by photon excitation from S_0_ (this might be weak), but also by conversions of other higher excited states (e.g., S_N_, T_N_) via multiple processes. As one of the most important parts in the photochemistry, therefore, the excited states investigated in this study are all located on S_1_.

**Figure 1:**
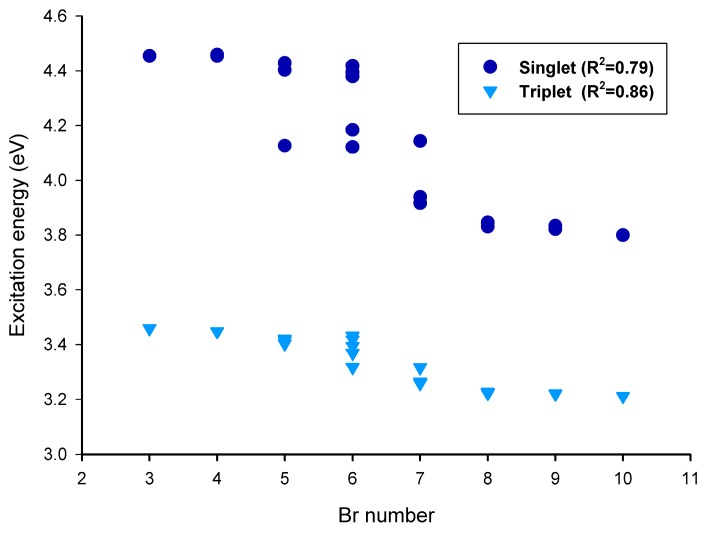
Correlation between lowest-lying excitation energy and Br number of the brominated diphenyl ether (BDE) congeners.

In this study, the obtained excitation energies of lowest-lying singlet (E_S1_) for the BDE congeners are between 3.79 to 4.46 eV, and the energy required for such photoexcitation corresponds to the ultraviolet light (327–278 nm), as shown in Table 1. Since PBDEs share the basic structure of diphenyl ether, the properties of BDE congeners will all depend on the bromination pattern. The E_S1_ values of the BDE congeners are linearly correlated negatively with the number of Br, as shown in Figure 1. For the three nona-BDEs under study, the E_S1_ values obtained are fairly close, while for the selected hepta- and hexa-BDEs, the E_S1_ values of the BDE congeners vary significantly.

**Table 1:** The lowest-lying singlet excitation energy for the BDE congeners and the weights of excited configurations.

Congener	E_S1_ (eV)	Wavelength (nm)	*f*	Assignment (H = HOMO, L = LUMO, L + 1 = LUMO + 1,* etc.*)
BDE-209	3.7996	326.3	0.0062	H→L (+74%); H-2→L + 1 (15%); H-3→L + 1 (7%)
BDE-208	3.8262	324.0	0.0022	H-1→L (+45%); H→L (+44%); H-3→L (7%)
BDE-207	3.8218	324.4	0.0037	H→L (+44%); H-1→L (42%); H-2→L (+10%)
BDE-206	3.8336	323.4	0.0026	H-1→L (+51%); H→L (+38%); H-3→L (6%)
BDE-203	3.846	322.4	0.0024	H-1→L (+70%); H→L (+23%)
BDE-196	3.8309	323.6	0.0029	H→L (+46%); H-1→L (36%); H-2→L (+7%)
BDE-190	3.9397	314.7	0.0001	H-1→L (+83%); H→L (14%)
BDE-183	4.1444	299.2	0.0012	H-1→L (+58%); H→L (36%)
BDE-181	3.9175	316.5	0.0025	H-1→L (+48%); H→L (+34%); H-2→L (+14%)
BDE-155	4.3949	282.1	0.0204	H→L (+83%); H-1→L + 1 (9%)
BDE-154	4.3799	283.1	0.0001	H→L + 2 (+42%); H→L + 1 (37%); H→L + 4 (18%)
BDE-153	4.4181	280.6	0.0025	H→L + 1 (+57%); H→L (16%); H→L + 3 (+8%)
BDE-139	4.1221	300.8	0.0015	H-1→L (+49%); H→L (+46%)
BDE-138	4.1844	296.3	0.0036	H→L (+79%); H-1→L (+15%)
BDE-100	4.4286	280.0	0.0121	H→L (+64%); H-1→L (+29%)
BDE-99	4.4037	281.6	0.0013	H→L + 1 (+73%); H→L (15%)
BDE-85	4.1269	300.4	0.0026	H→L (+84%); H-1→L (+12%)
BDE-77	4.4590	278.1	0.0183	H→L (+79%)
BDE-47	4.4543	278.4	0.0197	H→L (+87%); H-2→L + 1 (+6%)
BDE-28	4.4539	278.4	0.0204	H→L (+88%)

**Figure 2:**
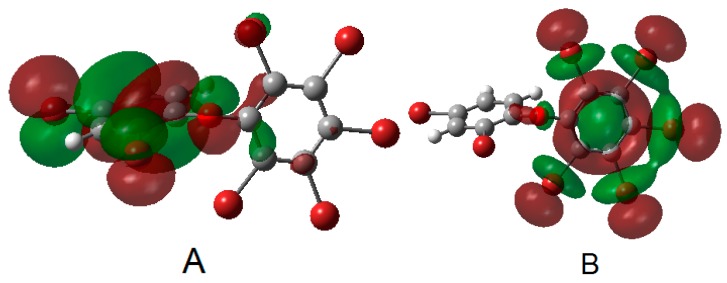
Frontier orbitals of BDE-181 (**A**: HOMO; **B**: LUMO) (iso-surface value = 0.02, arbitrary unit).

For ten of the selected twenty BDE congeners (Table 1), the electron excitation from highest occupied molecular orbital (HOMO) to lowest unoccupied molecular orbital (LUMO) made the biggest contribution to S0→S1 excitation. The HOMOs for most of the BDE congeners were found to be π-based, and preferred to spread mainly over the aromatic ring of fewer bromine substituents (especially for the lower brominated BDE congeners). Conversely, the LUMOs for the BDE congeners under study generally have the σ* character, and were mainly located on the phenyl group which has relatively more bromine substituents (e.g., BDE-181, Figure 2). This should be caused by the electron-withdrawing effects of the bromine atoms on the two phenyl rings. According to the assumption of Kasha’s rule, higher electronic states (S_N_) should decay exclusively via internal conversion to the lowest excited state in a solvent where collisions are common and energy dissipation is fast [33]. Therefore, after excitation, the molecules could convert to S1whichproceed from π orbital to σ* orbital showing the characteristics of the coherent HOMO-LUMO electronic transition. A similar phenomenon was found in the photoreductive debromination of a halogen-binding-based complex between decabromodiphenyl ether (BDE-209) and carboxylate under visible light irradiation [24]. Unlike other halogenated aromatic compounds, such as polychlorinated dibenzo-*p*-dioxins (PCDDs) and polychlorinated dibenzofurans (PCDFs), the conjugated system of the two aromatic rings for PBDEs is relatively weak. For the BDE congeners not symmetrically brominated on the two aromatic rings, the phenyl group with more Br substituents becomes more electron-deficient than the other one. Therefore, the electronic excitation from HOMO to LUMO of PBDEs would make the electron of electronic-rich ring transferred to the electron-deficient ring, resulting in a situation similar to the BDE anionic species which captured an additional electron in the LUMO [34,35,36]. Consequently, this transition of electrons into these anti-bond orbitals will reduce the C–Br bond order, and the C–Br bonds become weakened and easy to break in the excited states of BDE congeners. This contribution of S1 to the photolysis of PBDEs could be supported by previous theoretical calculations, the structure of PBDEs in singlet excited state and the structure of PBDE anions have the same geometrical characteristic,* i.e.*, the significant lengthening and out-of-plane bending of C–Br bonds [28,37,38]. Zeng* et al.* [39] also found the major debromination products of PBDEs have a greater similarity between photodebromination and Fe0 reduction and the linear relationships are significantly high between the LUMO energies and the debromination rates of PBDEs by treatment using UV light or zero-valent iron. In addition, it can be inferred that with one aromatic ring having relatively more bromine substituents than the other one, the electronic excitation of this kind of BDE congeners might be relatively easy, and the photochemical debromination, according to the characteristics of S1, would prefer to occur on the benzene ring having a higher bromination. These assumptions could be in agreement with the results observed in the previous photodebromination experiments [40].

### 2.2 The Lowest-Lying Triplet Excitations for Selected BDE Congeners

The electron in the LUMO is easy to spin-reverse due to the heavy atom effect of Br, thus the singlet excited state of the brominated compounds is expected to undergo efficient intersystem crossing to form the triplet state [24,41,42]. The lowest-lying triplet excitation energy (E_T1_) (Table 2) of the BDE congeners is significantly correlated with the E_S1_ (*R*^2^ = 0.937). However, in comparison with the singlet excitation of the BDE congeners under study, the weights of excited configurations for their triplet excitation are totally different and relatively complex.

**Table 2:** The lowest-lying triplet excitation energy for the BDE congeners and positions of the bent C–Br bond for the optimized geometries.

Congener	E_T1_ (eV)	Wavelength (nm)	Position of the Bent C–Br Bond
BDE-209	3.2118	386.0	4 (para-position)
BDE-208	3.22	385.0	4 (para-position)
BDE-207	3.221	384.9	4 (para- position)
BDE-206	3.221	384.9	4 (para-position)
BDE-203	3.2274	384.1	4' (para-position)
BDE-196	3.2229	384.7	4 (para-position)
BDE-190	3.2642	379.8	4 (para- position)
BDE-183	3.3164	373.8	4' (para- position)
BDE-181	3.2591	380.4	3 (meta-position)
BDE-155	3.3942	365.3	2 (ortho-position)
BDE-154	3.4327	361.2	5 (meta-position)
BDE-153	3.418	362.7	2 (ortho-position)
BDE-139	3.3175	373.7	2' (ortho-position)
BDE-138	3.3693	368.0	2 (ortho-position)
BDE-100	3.4028	364.4	2 (ortho-position)
BDE-99	3.4209	362.4	2 (ortho-position)
BDE-85	3.4145	363.11	2 (ortho-position)
BDE-77	3.4481	359.5	4 (para-position)
BDE-47	3.4479	359.6	2 (ortho-position)
BDE-28	3.4594	358.4	-^a^

^a^ bending of the C–Br bond was not observed in the present calculations.

**Figure 3:**
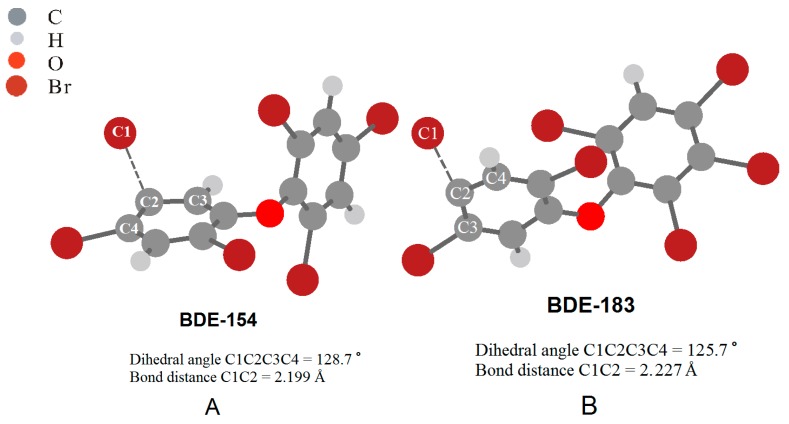
Visualized geometries of BDE-154 (**A**) and BDE-183 (**B**) in the triplet excited state.

In the present work, the DFT calculations (PCM/B3LYP/6-31G(d)) were employed to investigate the geometries of the lowest triplet excited states of the BDE congeners. Results show that the structures of the triplet excited states differ significantly from that of the BDE congeners in ground states. For the BDE congeners under study (except for BDE-28), one of the C–Br bonds at a specific position is bent out of the aromatic ring plane with the angles from around 50 to 70 degrees that increases with a decrease in the number of Br, as shown in Figure 3 (visualized geometries of the BDE-154 and BDE-183 in triplet excited state are taken as examples, and the others can be found in Appendix A). The degrees of this bond bending in the triplet excited states of PBDEs are significantly larger than those in their anionic states, probably indicating that the cleavage of C–Br bonds for PBDEs would occur more easily by excitation than by electron attachment [34,35]. In addition, the singly occupied molecular orbital (SOMO) of the triplet excited states for the BDE congeners were examined. The SOMO of the congeners (corresponding to the original HOMO in ground states) did not change significantly, leaving the π character. However, the SOMO + 1 (corresponding to the original LUMO in ground states) of the most BDEs is of the mixing characteristics of π and σ orbitals, or twisted σ character, which is believed to play an important role in the dissociation of halogen atoms from the halogenated compounds (e.g., PCDDs and PBDEs) induced by electron attachment [35,36]. From Table 2, we can see that the bending of C–Br bonds in the triplet excited states occurred preferably at the para-position for higher brominated BDE congeners and at the ortho-position for lower brominated BDE congeners. Similar results can also be observed in the corresponding gas-phase calculations. This is in agreement with the previous report that photodebromination of BDE-209 occurred mostly at the para-position [43]. Fang* et al.* [40] also reported that the photo-reactivity of bromines at various positions of phenyl rings decreased in the following order: ortho > para for lower brominated PBDEs (e.g., BDE-47), while for higher brominated congeners, the regioselectivity of photodebromination was not significant.

In addition, the geometry optimizations with higher multiplicity (e.g., five and seven) were performed, showing a bending of multiple C–Br bonds of the BDE congeners. This indicated the possibility of elimination of more than one Br atom in the photodebromination by multi photon excitation.

### 2.3. Quantitative Structure-Property Relationship (QSPR) between the Photodebromination Rate Constants and Molecular Descriptors

#### 2.3.1. Establishment of the Partial Least Squares Regression (PLSR), Principal Component Analysis-Multiple Linear Regression Analysis (PCA-MLR), and Back Propagation Artificial Neural Network (BP-ANN) Models

Photodebromination rate constants of the BDE congeners by UV light and sunlight used in this QSPR study were collected from Eriksson* et al.* [19] (*n* = 15, in the mixed solvent (methanol:water, 80:20)) and Wei* et al.* [32] (*n* = 13, in hexane), respectively. The original kinetic data were then randomly divided into two parts called training set and test set, for the model building and validation, respectively. Six molecular parameterscalculated at the PCM/B3LYP/6-311++G(d,p) level were chosen as the descriptor variables (as shown in Table S1), including E_T1_, E_S1_, E_LUMO_, E_HOMO_, HOMO-LUMO gap and the number of Br, which are popular descriptors for the stability and reactivity of molecules in theoretical and computational chemistry [44,45,46] and are important molecular parameters used to investigate the photolytic reactions [26,47,48].

The Pearson correlation analysis showed a high intercorrelation among the six independent variables (Table S2). For the establishment of the PLSR model, the first latent variable was obtained and then used for the regression. For the PCA-MLR approach, one principal component (PC1) was extracted from the original data (Table S3) and was taken as the variable to establish the linear regression equation. For the BP-ANN model, a multilayer feed-forward (MLFF) neural network with a back-propagation (BP) supervised learning method was selected with different architectures composed of different numbers of neurons in the hidden layer. The tangent sigmoid function as the transfer function was carried out by including input neurons as the six molecular descriptors related to logarithm of the debromination rate constants of the BDE congeners (log k). Gradient descent method was used to train the network. The training goal was set by 0.02. The learning rate was set by 0.05. Before training, all values of the inputted molecular descriptors were normalized between 0.1 and 0.9 using the following equation:

(3) Normalized value=0.1+(original value−min)/(max−min)×(0.9−0.1)

where “max” and “min” represent the maximum and minimum values, respectively, for a specific descriptor. Due to the random initialization of initial connection weight values at the start of the training process, the predicted results of the BP-ANN might vary slightly. Therefore, the final performance of the model was evaluated based on the average results from the simulations with ten repetitions.

There are several theories about determining the optimal network size used in statistical inference [49,50,51,52]. One of the theories indicates that the number of parameters in the network should be smaller than the number of observations. Although these rule-of-thumb methods aim to prevent overfitting and are useful in some cases, they are not considered to be true and reliable always as the optimal number of parameters is likely to depend on other factors, e.g., the distribution of the data points and the amount of noise [52,53,54]. A model which has been overfit will generally have poor predictive performance, as it can exaggerate minor fluctuations in the data. In general, with a fixed number of training patterns, overfitting can occur when the model has too many degrees of freedom. Considering the rather small number of observations obtained for the training sets (11 observations for the log *k*_UV_ and 9 observations for the log *k*_sun_), the maximum neurons in the hidden layer in this study was limited to 4 neurons (30 connections). The required number of hidden neurons was optimized by an iterative process, and the predictive ability of the network was separately validated by the test set (*n* = 4) [55,56].

In addition, the artificial neural networks are generally presented as systems of interconnected “neurons” which can compute values from inputs, and thus both the R factor and other evaluating indicators such as standard statistical error are invalid for effective evaluation of the fitting performance. Therefore, for comparative purposes, the values of RMSE (root mean squared error) were employed so that the three models (PLSR, PCA-MLR, and ANN) may be compared using their RMSE value as a measure of how well they explain a given set of observations. The RMSE can be calculated by Equation (4).

(4) $$RMSE=\sqrt{\frac{\sum {\left(\mathrm{observed}-\mathrm{predicted}\right)}^{2}}{n}}$$

#### 2.3.2. Performance of the QSPR Models

As shown in Table 3, the performance of the PLSR and PCA-MLR models are similar solely considering the data in the training set, since the *R*^2^*Y* in PLSR and *R*^2^ in PCA-MLR are fairly close with the values around 0.91 (*n* =15) and 0.83 (*n* = 13) for log *k*_UV_ and log *k*_sun_, respectively. For the training sets (Table 4), both the PLSR and PCA-MLR models presented the relatively large predicted values of the hexa-BDE congener (BDE-139), albeit BDE-139 has an experimental log *k*_UV_ close to that of the other hexa-BDEs (BDE-138, BDE-154, and BDE-155). Similarlytothe training set for log *k*_sun_, the PLSR and PCA-MLR models both overestimated the log *k*_sun_ of BDE-100, which actually has a relatively low reactivity in comparison with the other penta-BDEs (BDE-85 and BDE-99). These deviated cases might be caused by the inadequate utilization of the descriptor variables during the component extraction as the weights of the variables were estimated based on the linear transition; however, the impact of the molecular parameters on the debromination reactivity of the compounds might not follow this mode. Therefore, in the test set, we can also see that the PLS and PCA-MLR models both give the log *k*_UV_ values of BDE-183 (hepta-BDE) with relatively large errors, compared with the prediction results for the other congeners in the test set. Clearly, the RMSE values calculated with the PCA-MLR model are smaller than those calculated with the PLSR model.

**Table 3:** Fitting results of the models established by partial least squares regression (PLSR), principal component analysis-multiple linear regression analysis (PCA-MLR) methods.

Data	PLSR	PCA-MLR
*training set*		
Log *k*_UV_ (*n* = 11)	*RMSE* = 0.300	*RMSE* = 0.232
	*R*^2^*X* = 0.901	*R*^2^ = 0.920
	*R*^2^*Y* = 0.891	*F* = 103.452
	*Q*^2^ = 0.879	*Sig.* = 0.000
Log *k*_sun_ (*n* = 9)	*RMSE* = 0.276	*RMSE* = 0.275
	*R*^2^*X* = 0.901	*R*^2^ = 0.838
	*R*^2^*Y* = 0.835	*F* = 36.252
	*Q*^2^ = 0.804	*Sig.* = 0.001
*test set*		
Log *k*_UV_ (*n* = 4)	*RMSE* = 0.32	*RMSE* = 0.231
Log *k*_sun_ (*n* = 4)	*RMSE* = 0.202	*RMSE* = 0.185

**Table 4:** Predicted results of the quantitative structure-property relationship (QSPR) models by PLSR, PCA-MLR and BP-ANN (4 neurons in hidden layer) using the reported logarithm of the debromination rate constants by UV light (log *k*_UV_) [19].

Congener		PLSR *^a^*	PCA-MLR *^a^*	BP-ANN *^b^*	log *k*_UV_
*training set*	BDE-209	−3.71 ± 0.11	−3.80 ± 0.13	−3.44 ± 0.09	−3.40
	BDE-208	−3.87 ± 0.1	−3.96 ± 0.12	−3.91 ± 0.11	−3.77
	BDE-206	−3.89 ± 0.1	−3.99 ± 0.11	−3.95 ± 0.04	−4.08
	BDE-203	−4.01 ± 0.09	−4.12 ± 0.11	−4.33 ± 0.07	−4.43
	BDE-190	−4.24 ± 0.08	−4.37 ± 0.09	−4.59 ± 0.04	−4.52
	BDE-155	−5.3 ± 0.07	−5.43 ± 0.10	−5.31 ± 0.08	−5.39
	BDE-154	−5.47 ± 0.08	−5.60 ± 0.11	−5.50 ± 0.13	−5.48
	BDE-139	−4.83±0.07	−4.96 ± 0.08	−5.31 ± 0.07	−5.40
	BDE-138	−5.15 ± 0.07	−5.30 ± 0.09	−5.40 ± 0.11	−5.21
	BDE-99	−5.6 ± 0.09	−5.75 ± 0.12	−5.67 ± 0.06	−5.52
	BDE-47	−5.9 ± 0.11	−6.08 ± 0.14	−5.96 ± 0.08	−6.16
*test set*	BDE-207	−3.88 ± 0.10	−3.98 ± 0.12	−3.92 ± 0.05	−3.72
	BDE-183	−4.73 ± 0.07	−4.85 ± 0.08	−5.06 ± 0.10	−5.17
	BDE-181	−4.2 ± 0.08	−4.33 ± 0.09	−4.61 ± 0.06	−4.50
	BDE-77	−5.9 ± 0.11	−6.08 ± 0.14	−5.96 ± 0.08	−6.22
*prediction*	BDE-196	−3.95 ± 0.10	−4.05 ± 0.11	−4.03 ± 0.12	-
	BDE-153	−5.43 ± 0.08	−5.57 ± 0.10	−5.52 ± 0.09	-
	BDE-100	−5.54 ± 0.09	−5.68 ± 0.11	−5.59 ± 0.20	-
	BDE-85	−5.30 ± 0.08	−5.46 ± 0.10	−5.41 ± 0.14	-
	BDE-28	−6.10 ± 0.12	−6.29 ± 0.16	−6.01 ± 0.15	-

*^a^* value ± standard error, *a* = 0.05; *^b^* value ± standard deviation, *n* = 10.

In the ANN models (as shown in Table 5), the best mean training error in the test set occurs at four and three (four) hidden neurons for log *k*_UV_ and log *k*_sun_, respectively. The generalization error is fairly small for all networks with the largest RMSE value of 0.27 for the architecture (6:1:1, log *k*_UV_). The underfitting is significant for the prediction of log *k*_UV_ when the number of the hidden neurons is smaller than two. For the prediction of log *k*_sun_, the difference of the RMSE values in the test set is slight in the four network architectures.

In order to minimize the parameters, the number of the neurons in hidden layer should thus be minimal (the best choice is only one neuron). Nevertheless, the prediction results for the test sets are unsatisfactory for the BP-ANN models with one neuron in the hidden layer, which might be caused by underfitting (Table 5). Thus, we chose four neurons for log *k*_UV_ and three neurons for log *k*_sun_ in the hidden layer for BP-ANN model. The predictions for the test set by the BP-ANN model are considerably better than those by the PLSR and PCA-MLR models for log *k*_UV_, but slightly poorer than those by the two models for log *k*_sun_.

Although the numbers of parameters (weights or connections) of the networks in three cases of this study are larger than the sample size, the validation of the networks by the test set seemed fairly good. An explanation might be that the extra degrees of freedom can aid convergence, and successive pruning and retraining of a larger network may arrive at a network with similar size or the smaller size networks but with improved training results [54,57]. In a large network, there may be many solutions which fit the training data well that will not generalize well; we therefore list herein the predicted results of each BDE congener in the test set by the BP-ANN models (with the optimized number of hidden layer neurons), PLSR, and PCA-MLR models for the purpose of comparison (as shown in Table 4 and Table 6).

**Table 5:** RMSE (root mean squared error) values for the optimization of the number of hidden layer neurons in BP-ANN.

Hidden Layer Neurons	1	2	3	4
Weights	7	14	21	28
Performance for log *k*_UV_				
Training (*n* = 11)	0.165	0.160	0.136	0.124
Test (*n* = 4)	0.270	0.268	0.216	0.182
Performance for log *k*_sun_				
Training (*n* = 9)	0.179	0.166	0.171	0.182
Test (*n* = 4)	0.240	0.251	0.216	0.216

**Table 6:** Predicted results of the QSPR models by PLSR, PCA-MLR and BP-ANN (3 neurons in hidden layer) using the reported logarithm of the debromination rate constants by sunlight (log *k*_sun_) [32].

Congener		PLSR *^a^*	PCA-MLR *^a^*	BP-ANN *^b^*	log *k*_sun_
*training set*	BDE-209	−3.3 ± 0.12	−3.26 ± 0.18	−3.27 ± 0.10	−3.24
	BDE-206	−3.44 ± 0.11	−3.39 ± 0.17	−3.43 ± 0.08	−3.43
	BDE-196	−3.50± 0.1	−3.44 ± 0.16	−3.49 ± 0.08	−3.48
	BDE-183	−4.07 ± 0.07	−4.03 ± 0.11	−4.04 ± 0.15	−4.10
	BDE-153	−4.58 ± 0.08	−4.55 ± 0.12	−4.38 ± 0.09	−4.23
	BDE-100	−4.67 ± 0.09	−4.63 ± 0.13	−4.83 ± 0.12	−5.23
	BDE-99	−4.71 ± 0.09	−4.68 ± 0.13	−4.57 ± 0.16	−4.30
	BDE-85	−4.52 ± 0.08	−4.47 ± 0.11	−4.26 ± 0.03	−4.27
	BDE-28	−5.13 ± 0.13	−5.08 ± 0.18	−5.24 ± 0.06	−5.24
*test set*	BDE-208	−3.42 ± 0.11	−3.37 ± 0.17	−3.41 ± 0.10	−3.18
	BDE-207	−3.44 ± 0.11	−3.39 ± 0.17	−3.41 ± 0.13	−3.19
	BDE-154	−4.60 ± 0.08	−4.57 ± 0.12	−4.21 ± 0.21	−4.51
	BDE-47	−4.97 ± 0.11	−4.92 ± 0.16	−5.14 ± 0.18	−5.16
*predictions*	BDE-203	−3.56 ± 0.10	−3.49 ± 0.15	−3.55 ± 0.23	-
	BDE-190	−3.75 ± 0.08	−3.67 ± 0.13	−3.80 ± 0.60	-
	BDE-181	−3.72 ± 0.09	−3.65 ± 0.14	−3.78 ± 0.52	-
	BDE-155	−4.47 ± 0.08	−4.45 ± 0.11	−4.32 ± 0.21	-
	BDE-139	−4.16 ± 0.07	−4.11 ± 0.10	−4.31 ± 0.23	-
	BDE-138	−4.40 ± 0.07	−4.35 ± 0.11	−4.43 ± 0.27	-
	BDE-77	−4.97 ± 0.11	−4.92 ± 0.16	−5.12 ± 0.21	-

*^a^* value ± standard error, *a* = 0.05; *^b^* value ± standard deviation, *n* = 10.

In summary, the predicted orders of the photodebromination reactivities for the congeners by all models are very consistent with the observed trends: BDE-207 > BDE-183 > BDE-181 > BDE-77 for UV debromination and BDE-208 > BDE-207 > BDE-154 > BDE-47 for sunlight debromination. It is noteworthy that the log *k*_UV_ values between BDE-183 and BDE-181 which have the same Br number (hepta-) but different bromination patterns were successfully predicted by the three methods, especially by BP-ANN. Since one of the aromatic rings for BDE-181 is fully brominated, the photodebromination reactivity of BDE-181 is reasonably higher than that of BDE-183, which has a relatively high symmetry of the bromination pattern on its two phenyl rings. The predictions of BDE-183 obtained with all BP-ANN models are significantly better than those made with PLSR and PCA-MLR models, exhibiting the nonlinear fitting ability of ANN. No significant difference was found between the prediction results given by BP-ANN models of different architecture (Tables S4 and S5).

In addition, a significant correlation was found between the log *k*_UV_ and log *k*_SUN_ (*R*^2^ = 0.95, *n* = 8), indicating that these two types of reactions are of clear similarity (initiated by the electron excitation or intermolecular electron transfer). However, the kinetic data obtained under natural sunlight irradiation for some congeners (e.g., BDE-298, BDE-207, and BDE-100) seemed to be abnormal (Table 6) according to the commonly accepted knowledge that the photochemical reactivity of PBDEs increased with an increasing number of bromines [40,43]. Two other earlier reports also supported the low photoreactivity of BDE-100, showing that under UV irradiation, this congener has the log *k* value similar to or even lower than BDE-28 [27,40]. The HOMO-LUMO gap is a popular molecular descriptor used to describe the reactivity of molecules, and this is also an approximation of the hardness of the system. In some systems, the HOMO-LUMO gap can be close to the excitation energy; however, they may not be identical after a comparison of several cases in this study. We examined the six selected variables and observed that the most possible factor accounting for the relatively low photoreactivity of BDE-100 might be the E_S1_, since this parameter of BDE-100 is distinctively higher than that of the other penta-BDEs, and close to that of the tetra-BDEs in this work. And the HOMO-LUMO gap value of BDE-100 seemed not special and just ranked in the middle of that of the other penta-BDEs. The three methods employed in this work, including PLSR, PCA-MLR and ANN, are all effective tools which are good at digging valuable information and dealing with this kind of problem. However, only the BP-ANN models in this study could distinctively identify the relatively low photoreactivity of BDE-100 in comparison with the other penta-BDEs (Table 5 and Table S6). In addition, the higher photoreactivities of the nona-BDEs than BDE-209 under sunlight is hard to explain by the models in this study. More molecular descriptors as well as the experimental data of similar cases for other BDE congeners are expected to help better understand the reasons behind this kind of “abnormal” low reactivity of some BDE congeners, and this still needs further research.

Since it is difficult to evaluate the variable importance in the PCA-MLR and BP-ANN models, the variable importance was evaluated only in the PLSR model. Results showed that, in the PLSR model, E_S1, _E_T1_ and number of Br are the two most important variables with the largest VIP (variable importance in the projection) values. It is reasonable that the process of excitation played an important role in the photodebromination of PBDEs [25]. In the debromination experiments of PBDEs, the intensities of the light emission (which directly affected the photoreaction rate) from artificial sources [30,40] or sunlight [58] are in general not permanent but increase with the increasing wavelength in the range of the PBDEs’ excitation wavelength. Thus, for the BDE congeners, photo-excitation at a longer wavelength would occur more easily. In addition, it is well known that the reactivity of PBDEs increases with the increase in the degree of bromination. For PBDEs, the Br number used in these models can also be viewed as the representation of molecular weights or the rough total energy. We observed that the simple linear correlations between the Br number and log *k* have high *R*^2^ values (0.95 for log *k*_UV_ and 0.88 for log *k*_SUN_), which were even better than those in the PCA-MLR models. However, only using the number of Br as the descriptor variable cannot distinguish the difference between the BDE congeners if they have the same Br number. It is well known that the photodebromination rates for BDE congeners depend not only on their degree of bromination but also on the bromination pattern. Bromination pattern significantly affects the conformation and the degree of conjugation of the PBDE molecules. The latter one is related to the molecular orbital energy and determines the actual energy difference between the ground and excited states. When the other five molecular descriptors were added in the dataset for the QSPR study, the established models were able to approximately represent (or quantify) the pattern of bromination of the BDE congeners as well as their reactivity. Therefore, some kinds of topological descriptors concerning the molecular structure might be worth trying for the QSPR study in the future [59,60].

## 3. Experimental Section

All quantum-chemical calculations were performed using Gaussian03 program suite [61] with GaussView 4.1 used as the molecular modeling system for constructing and visualizing the results of the calculations. To facilitate our calculations, methanol was chosen as the solvent included in our calculations using the polarized continuum model (PCM), considering that the observed photodegradation pathways of the BDEs seemed to be consistent among the different solvent matrices tested (toluene, methanol, and THF) [62] and the photodebromination rates of the congeners were highly correlated in different solvents (methanol/water (80:20) solution, methanol, THF, and hexane) [19,32]. Prior to calculating the excited states, all molecules were geometry-optimized in their ground state at the PCM/B3LYP/6-31G(d) level of theory. The minimum geometries were confirmed by a frequency analysis. Then, the TD-DFT was employed to calculate the vertical excitation energy of the BDE congeners at the PCM/B3LYP/6-311++G(d,p) level, including the lowest-lying singlet and the lowest-lying triplet excitation energies. The PCM/B3LYP/6-31G(d) calculations were employed to investigate the structure of triplet excited states by setting the multiplicity to triplet. Assignment of the singlet excited states was conducted with the use of SWizard 4.6 program (Gorelsky SI, Ottawa, ON, Canada). The PLSR was carried out by SIMCA-P software (demo version 11.5.0.0, Umetrics AB, San Jose, CA, USA). The PCA-MLR was undertaken with the use of PASW (Version 18.0.0, SPSS Inc., Chicago, IL, USA). The BP-ANN was built with the Neural Network Toolbox of MATLAB software (2010a) (the MathWorks, Inc., Natick, MA, USA).

The IUPAC names and the congener numbers of the 2-selected PBDEs in this study are listed in Table 7.

**Table 7:** IUPAC names and the congener numbers of the twenty selected BDE congeners.

No.	IUPAC Name
BDE-209	Deca-bromodiphenyl ether
BDE-208	2,2',3,3',4,5,5',6, 6'- nona-bromodiphenyl ether
BDE-207	2,2',3,3',4,4',5,6,6'-nona-bromodiphenyl ether
BDE-206	2,2',3,3',4,4',5,5',6-nona-bromodiphenyl ether
BDE-203	2,2',3,4,4',5,5',6-octa-bromodiphenyl ether
BDE-196	2,2',3,3',4,4',5,6'-octa-bromodiphenyl ether
BDE-190	2,3,3',4,4',5,6-hepta-bromodiphenyl ether
BDE-183	2,2',3,4,4',5',6-hepta-bromodiphenyl ether
BDE-181	2,2',3,4,4',5,6-hepta-bromodiphenyl ether
BDE-155	2,2',4,4',6,6'-hexa-bromodiphenyl ether
BDE-154	2,2',4,4',5,6'-hexa-bromodiphenyl ether
BDE-153	2,2',4,4',5,5'-hexa-bromodiphenyl ether
BDE-139	2,2',3,4,4',6-hexa-bromodiphenyl ether
BDE-138	2,2',3,4,4',5'-hexa-bromodiphenyl ether
BDE-100	2,2',4,4',6-penta-bromodiphenyl ether
BDE-99	2,2',4,4',5-penta-bromodiphenyl ether
BDE-85	2,2',3,4,4'-penta-bromodiphenyl ether
BDE-77	3,3',4,4'-tetra-bromodiphenyl ether
BDE-47	2,2',4,4'-tetra-bromodiphenyl ether
BDE-28	2,4,4'-Tri-bromodiphenyl ether

## 4. Conclusions

In this study, both DFT and TD-DFT methods were employed to investigate the singlet excited states and triplet excited states of the 20selected BDE congeners. For most of the BDE congeners, the singlet excited state was initiated by the electron transfer from HOMO to LUMO, involving a π–σ* excitation. The electron density of the HOMO and LUMO differently located on the two phenyl rings of most BDE congeners showed that the electronic excitation of these compounds could make the electron of the electronic-rich ring transferred to the electron-deficient ring. Thus, the direct photochemical debromination would prefer to occur on the benzene ring of a higher bromination level. Structures in the triplet excited states of the BDE congeners differ notably from those in the ground states, with one of the specific C–Br bonds bending off the aromatic plane. This bond bending occurs preferably at the para-position for the higher brominated BDE congeners and at the ortho-position for the lower brominated congeners, being compatible with the experimentally observed photodebromination preference [39,43]. Results from the QSPR studies show that PLSR, PCA-MLR and ANN could satisfactorily predict the rate constants of the BDE photodebromination.

In comparison with other remediation methods for PBDE contamination, photodebromination has a unique advantage since solar energy is a kind of efficient and renewable energy source especially with the assistance of the photocatalyzers (e.g., TiO_2_ [63] and graphitic carbon nitride [64]). Thus, more studies should be conducted in this field to facilitate the improvement of remediation techniques (such as soil contamination remediation) and to better evaluate the potential risk of PBDEs.

## Supplementary Materials

Supplementary materials can be found at http://www.mdpi.com/1422-0067/16/01/1160/s1.
